# Supplementary material for: A dynamic charge-charge interaction modulates PP2A:B56 substrate recruitment
Source: eLife. 2020 Mar 20;9:e55966. doi: 10.7554/eLife.55966 (PMC7108865; doi:10.7554/eLife.55966)
Supplement: Transparent reporting form [file elife-55966-transrepform.docx]

***eLife’s* transparent reporting form**

We encourage authors to provide detailed information *within their submission* to facilitate the interpretation and replication of experiments. Authors can upload supporting documentation to indicate the use of appropriate reporting guidelines for health-related research (see [EQUATOR Network](http://www.equator-network.org/%20)), life science research (see the [BioSharing Information Resource](https://biosharing.org/)), or the [ARRIVE guidelines](http://www.plosbiology.org/article/info:doi/10.1371/journal.pbio.1000412) for reporting work involving animal research. Where applicable, authors should refer to any relevant reporting standards documents in this form.

If you have any questions, please consult our Journal Policies and/or contact us: [editorial@elifesciences.org](mailto:editorial@elifesciences.org).

**Sample-size estimation**

- You should state whether an appropriate sample size was computed when the study was being designed
- You should state the statistical method of sample size computation and any required assumptions
- If no explicit power analysis was used, you should describe how you decided what sample (replicate) size (number) to use

Please outline where this information can be found within the submission (e.g., sections or figure legends), or explain why this information doesn’t apply to your submission:

n/a

**Replicates**

- You should report how often each experiment was performed
- You should include a definition of biological versus technical replication
- The data obtained should be provided and sufficient information should be provided to indicate the number of independent biological and/or technical replicates
- If you encountered any outliers, you should describe how these were handled
- Criteria for exclusion/inclusion of data should be clearly stated
- High-throughput sequence data should be uploaded before submission, with a private link for reviewers provided (these are available from both GEO and ArrayExpress)

Please outline where this information can be found within the submission (e.g., sections or figure legends), or explain why this information doesn’t apply to your submission:

**REPLICATES**

**ITC experiments:** The number of replicates reported in Table 1. The replicates reported are technical replicates.

**Mass Spectrometry experiments**: Pull-down experiments performed in triplicate (technical replicates: pull-downs performed three times. This information is included in the Methods.

**Western Blots.** Blots are representative images from at least two independent experiments. This information is included in the methods and in the figure legends

**Time-lapse**. Representative experiment is shown out of three independent experiments. This information is provided in the figure legend.

**Statistical reporting**

- Statistical analysis methods should be described and justified
- Raw data should be presented in figures whenever informative to do so (typically when N per group is less than 10)
- For each experiment, you should identify the statistical tests used, exact values of N, definitions of center, methods of multiple test correction, and dispersion and precision measures (e.g., mean, median, SD, SEM, confidence intervals; and, for the major substantive results, a measure of effect size (e.g., Pearson's r, Cohen's d)
- Report exact p-values wherever possible alongside the summary statistics and 95% confidence intervals. These should be reported for all key questions and not only when the p-value is less than 0.05.

Please outline where this information can be found within the submission (e.g., sections or figure legends), or explain why this information doesn’t apply to your submission:

**STATISTICAL REPORTING**

**ITC experiments**: Data analyzed using the ITC data processing software NITPIC , SEDPHAT and GUSSI, as described in the methods. The data reported in Table 1 represent the average and standard deviation, as described in the methods.

**Mass Spec experiments**: Statistical analysis of protein quantification carried out in Perseus by two-tailed student t-test. This information is found in the Methods.

**Western Blots.** Blots quantified using the Odyssey Sa Application software. Quantifications are indicated under the blots.

**Quantification of mitotic exit.** Circles represent single cells. The number of cells and median (red line) times are indicated from at a representative experiment. Three independent experiments were carried out. Mann-Whitney test was used to determine the p-values indicated. ∗∗∗∗ P < 0.0001; * P <0.05; ns, not significant. This information is included in the legend to Figure 4.

(For large datasets, or papers with a very large number of statistical tests, you may upload a single table file with tests, Ns, etc., with reference to sections in the manuscript.)

**Group allocation**

- Indicate how samples were allocated into experimental groups (in the case of clinical studies, please specify allocation to treatment method); if randomization was used, please also state if restricted randomization was applied
- Indicate if masking was used during group allocation, data collection and/or data analysis

Please outline where this information can be found within the submission (e.g., sections or figure legends), or explain why this information doesn’t apply to your submission:

n/a

**Additional data files (“source data”)**

- We encourage you to upload relevant additional data files, such as numerical data that are represented as a graph in a figure, or as a summary table
- Where provided, these should be in the most useful format, and they can be uploaded as “Source data” files linked to a main figure or table
- Include model definition files including the full list of parameters used
- Include code used for data analysis (e.g., R, MatLab)
- Avoid stating that data files are “available upon request”

***Please indicate the figures or tables for which source data files have been provided:***

1. Source data have been provided for Figures 1D/E and Figure 4E (the MS data).
2. Figure 3: All NMR chemical shifts have been deposited in the BioMagResBank (BMRB 27913). Atomic coordinates and structure factors have been deposited in the Protein Data Bank (6OYL, 6VRO). The mass spectrometry proteomics data have been deposited to the ProteomeXchange Consortium (Vizcaíno et al., 2014) through the PRIDE partner repository (PXD013886).
